# Genome-Wide Analysis of C2H2 Zinc Finger Gene Family and Its Response to Cold and Drought Stress in Sorghum [*Sorghum bicolor* (L.) Moench]

**DOI:** 10.3390/ijms23105571

**Published:** 2022-05-16

**Authors:** Huiying Cui, Jiaqi Chen, Mengjiao Liu, Hongzhi Zhang, Shuangxi Zhang, Dan Liu, Shaolin Chen

**Affiliations:** 1 College of Life Sciences, Northwest A&F University, Xianyang 712100, China; jqc@nwafu.edu.cn (J.C.); lmj@nwafu.edu.cn (M.L.); ZHANGHZ612@nwafu.edu.cn (H.Z.); joker@nwafu.edu.cn (S.Z.); xinongliudan@nwafu.edu.cn (D.L.); 2 Biomass Energy Center for Arid and Semi-Arid Lands, Northwest A&F University, Xianyang 712100, China

**Keywords:** C2H2, sorghum, gene family, phylogenetic analysis, stress responses

## Abstract

C2H2 zinc finger protein (C2H2-ZFP) is one of the most important transcription factor families in higher plants. In this study, a total of 145 *C2H2-ZFPs* was identified in *Sorghum bicolor* and randomly distributed on 10 chromosomes. Based on the phylogenetic tree, these zinc finger gene family members were divided into 11 clades, and the gene structure and motif composition of *SbC2H2-ZFPs* in the same clade were similar. *SbC2H2-ZFP* members located in the same clade contained similar intron/exon and motif patterns. Thirty-three tandem duplicated *SbC2H2-ZFPs* and 24 pairs of segmental duplicated genes were identified. Moreover, synteny analysis showed that sorghum had more collinear regions with monocotyledonous plants such as maize and rice than did dicotyledons such as soybean and Arabidopsis. Furthermore, we used quantitative RT-PCR (qRT-PCR) to analyze the expression of *C2H2-ZFPs* in different organs and demonstrated that the genes responded to cold and drought. For example, *Sobic.008G088842* might be activated by cold but is inhibited in drought in the stems and leaves. This work not only revealed an important expanded *C2H2-ZFP* gene family in *Sorghum bicolor* but also provides a research basis for determining the role of *C2H2-ZFPs* in sorghum development and abiotic stress resistance.

## 1. Introduction

The zinc finger protein family has evolved into a massive transcription factor family in higher plants [1]. Each zinc finger is approximately 23–30 amino acids in length, which is composed of cysteine and histidine combined with zinc ions through hydrogen bonds. It is well known that ZFP types are usually classified based on the number and position of cysteine and histidine residues, and ZFP can be divided into 10 subclasses, namely C2H2, C2HC, C2HC5, C2C2, CCCH, C3HC4, C4, C4HC3, C6, and C8 [2]. Among them, C2H2 zinc finger is one of the most general motifs in ZFP, which was initially reported in the African clawed frog (*Xenopus laevis*) transcription factor IIIA (TFIIIA) protein, so it is also called TFIIIA zinc finger protein [3]. Furthermore, its sequence feature is CX2-4CX12HX3-5H, where X stands for amino acid, C stands for cysteine, and H stands for histidine, while the number stands for the number of residues [4]. The number of C2H2 zinc fingers has great variability, ranging from one to dozens, which implies that the function of C2H2-ZFPs is very wide [5].

So far, more than 10 species of C2H2-ZFPs have been identified, including *Arabidopsis thaliana* (176), *Glycine max* (321), *Camellia sinensis* (134), *Medicago truncatula* (218), tomato (112), durum wheat (122), and rice (189) [6,7,8,9,10,11,12]. Because zinc fingers can bind to the main grooves of the DNA sequence, C2H2-ZFPs can affect DNA transcription, but the functions of C2H2-ZFPs are far more than activating or inhibiting transcription. In higher plants, they can also package RNA, assemble proteins, bind lipids, and participate in the regulation of apoptosis [13].

Studies show that C2H2-ZFPs have a wide range of effects on plant growth and development and resistance to abiotic stresses. In tomato, C2H2 zinc finger proteins H and Sh were found to be important transcription factors that controlled the initiation and elongation of type I and III multicellular trichomes [14]. At the same time, SlZFP6 was upregulated by protein H, which increased the density and length of tomato trichomes [15]. Apart from that, when the SUMOylation of the zinc finger transcription factor STOP1 was up-regulated, plants could enhance the resistance to aluminum stress in *Arabidopsis* [16]. Moreover, in *Medicago truncatula*, MtSUPERMAN could regulate compound inflorescence and flower development [17]. In addition, POPOVICH played a core part in the development of flower nectar stimulation in Aquilegia [18]. Furthermore, ectopic expression of MdZAT10 in *Arabidopsis* reduced the tolerance to drought stress [19].

Sweet sorghum (*Sorghum bicolor*) can be grown in most semi-arid regions of the world and has high sugar production and high photosynthetic conversion rate, so it is often used as a high energy crop and is planted all over the world [20]. The growth and development of sorghum is affected by various abiotic stresses, including drought stress and low temperature stress, which directly restrict the yield and quality of sorghum [21,22]. Although sorghum is drought-tolerant, saline–alkali-tolerant, and sensitive to low temperature, the mechanism is still unclear, and only a few C2H2 zinc finger genes have been identified in sorghum [23,24,25,26]. For example, overexpression of the *SbSTOP1* gene can increase sorghum resistance to aluminum stress by activating the transcription of a β-1,3-glucanase gene [25]. Moreover, four STOP1-like proteins (SbSTOP1a, SbSTOP1b, SbSTOP1c, and SbSTOP1d) have been identified to be associated with aluminum tolerance, and SbSTOP1 might function as a homodimer and/or heterodimer [24]. However, most C2H2-ZFPs have not been identified in sorghum. Therefore, the whole genome sequence and expression of SbC2H2-ZFPs were analyzed in this study.

A total of 145 C2H2-type ZFPs was identified in this research. These zinc finger gene family members were divided into 11 clades by the phylogenetic tree. The SbC2H2-ZFP members located in the same clade contained similar intron/exon and motif patterns. Tandem repeats contributed more to the increase in C2H2-ZFP membership in sorghum than segmental repeats. Almost all C2H2-ZFPs of clades were expressed in roots, stems, and leaves, and we used qRT-PCR to determine the responses of C2H2-ZFPs to cold and drought stress. Our results enriched the knowledge of structural information, evolutionary relationship, and the expression patterns of sorghum C2H2-ZFPs and provided a basis for further studies on C2H2-ZFPs in the growth and stress resistance of sorghum.

## 2. Results

### 2.1. Identification of C2H2-ZFP Genes in S. bicolor

After removing genes containing multiple transcripts, there were found to be only 109 C2H2-ZFPs listed in the sorghum transcription factor database (http://planttfdb.gao-lab.org/family.php?sp=Sbi&fam=C2H2, accessed on 5 September 2021). Given there were 167 C2H2-ZFPs reported in *Arabidopsis*, we thought that there might be more C2H2-ZFPs in the *S. bicolor* genome. To identify all C2H2-ZFP members in *S. bicolor*, the hidden Markov model (HMM) profiles (PF00096, PF13894, PF13912, PF18414, PF16622, and PF18658) from the Pfam database were used for searching *SbC2H2-ZFP* genes (http://pfam.xfam.org/, accessed on 5 September 2021). The *SbC2H2-ZFP* genes were identified through alignment against *Arabidopsis C2H2-ZFP* sequences (e-value < 0.01). Furthermore, the sequences lacking the C2H2-zinc finger motif were removed based on MEME (https://meme-suite.org/meme/tools/meme, accessed on 25 September 2021) search (e-value < 0.05). Eventually, a total of 145 *C2H2-ZFP* genes was identified in *S. bicolor* (Table S1). *Sobic.002G116600* has a minimum molecular weight (Mw) of 10.82 kDa, while *Sobic.004G265500* has a maximum molecular weight of 178.48 kDa. Moreover, isoelectric point (pI) values of the *SbC2H2-ZFPs* were between 4.54 and 10.25, 47.65% of which were over 7.0.

### 2.2. Phylogenetic Analysis and Classification of SbC2H2-ZFPs

**Figure 1:**
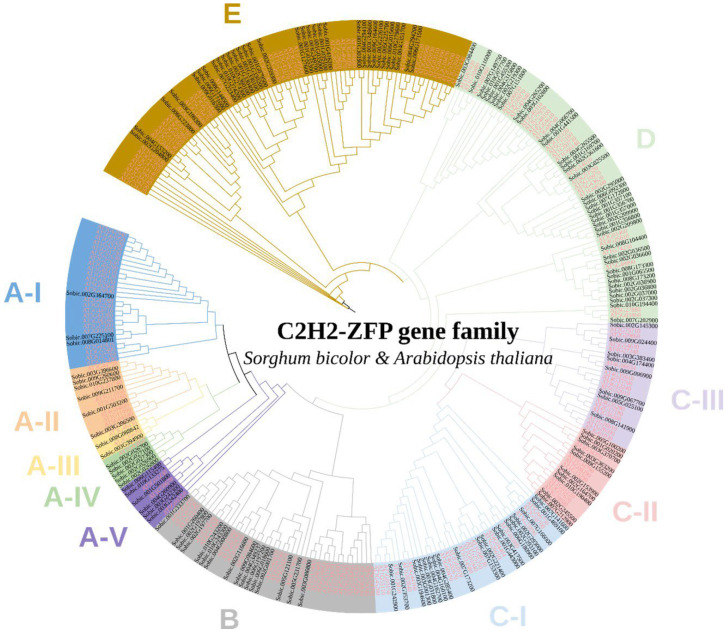
Phylogenetic tree of C2H2-ZFP members between *Arabidopsis thaliana* and *Sorghum bicolor*. The tree was constructed with the maximum likelihood (ML) method. The ranges and branches of the circular tree in 11 clades were marked using different colors. C2H2-ZFP proteins from *Arabidopsis* and *Sorghum bicolor* have the prefix “AT” and “Sobic”, respectively.

To predict functions of *SbC2H2-ZFPs*, a phylogenetic tree containing both *A. thaliana* and *S. bicolor* C2H2-ZFPs was built by the maximum likelihood (ML) method. Based on the sequence similarity and topology, SbC2H2-ZFPs were divided into five clades containing clade A, clade B, clade C, clade D, and clade E (Figure 1). Twenty-two SbZFPs and 37 AtZFPs belonged to clade A, 16 SbZFPs and 27 AtZFPs were part of clade B, 37 SbZFPs and 41 AtZFPs were assigned to clade C, 37 SbZFPs and 26 AtZFPs were grouped into clade D, and 33 SbZFPs and 42 AtZFPs were sections of clade E.

Clade A was divided into five subclades—clade A-I, A-II, A-III, A-IV, and A-V. Clade A-I contained many ubiquitin carboxyl-terminal hydrolase-related genes such as AT5G61940, AT1G52430, and AT5G02660, as well as the known MAZ1 (AT5G15480), which regulates intine formation and the exine pattern in Arabidopsis [27]. In addition, ELF6 (AT5G04240) in clade A-II and FIS2 (AT2G35670) in clade A-III play roles in pollination and flowering [28,29]. Clade B contained REIL2 (AT2G24500) responding to cold stress and URO (AT3G23140) relevant to IAA homeostasis [30].

In addition, clade C was divided into three subclades—clade C-I, C-II, and C-III. Clade C-I consisted of indeterminate-domain (IDD)-type C2H2-ZFPs, such as BIB (AT3G45260), IDD7 (AT1G55110), IDD11 (AT3G13810), IDD12 (AT4G02670), and IDD1 (AT5G66730). IDD-type C2H2-ZFPs play roles in a variety of plant growth and development processes, including root development, flowering, seed maturation, leaf growth, hormone regulation, and defense against pathogens [31]. Additionally, clade C-II contained STOP and WIP-type C2H2-ZFPs. STOP1 (At1g34370) protects plants against acidic soils [32]. WIP-type AtC2H2-ZFPs, including WIP1 (AT1G34790) and WIP2 (AT3G57670), regulate endothelium differentiation, pollen tube development, and leaf vasculature growth in plants [33,34]. Apart from that, DAZ1 (AT2G17180) and DAZ2 (AT4G35280), which were in clade C-III, are involved in pollen fertility.

What is more, ZFP6 (AT1G67030), ZFP5 (AT1G10480), GIS (AT3G58070), GIS2 (AT5G06650), and ZFP8 (AT2G41940) contribute to trichome branching in clade D. Moreover, clade E included ZAT6 (AT5G04340), relevant to cold stress, and ZAT10 (AT1G27730), responding to salt tolerance [35,36]. Moreover, ZAT7 (At3g46090), the EAR motif of the AtC2H2-type zinc finger protein, inhibits WRKY70 expression under salt stress [37].

### 2.3. Gene Features and Conserved Motifs of SbC2H2-ZFPs

**Figure 2:**
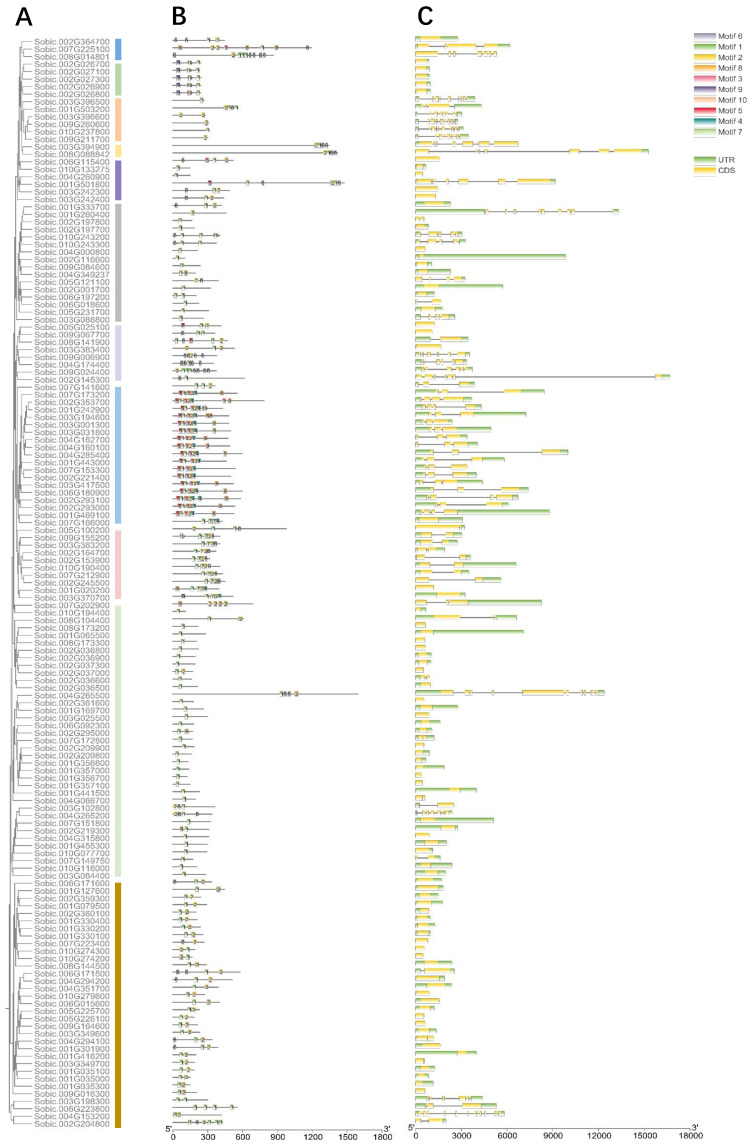
Motif distributions and gene-structure analysis of *SbC2H2-ZFP* genes. (**A**) The phylogenetic tree was built by the ML method with a bootstrap value of 1000. (**B**) The conserved motifs in *SbC2H2-ZFP* proteins (1–10) are in different colors. The black lines represent relative protein lengths. (**C**) Exons, introns, and untranslated region (UTR) are represented by yellow rectangles, gray lines, and green rectangles, respectively. The length of *SbC2H2-ZFPs* is shown below.

According to phylogenetic analysis in Figure 2A, 145 SbC2H2-ZFPs were divided into 11 subclasses (Figure 2A). A total of 145 SbC2H2-ZFPs protein sequences was analyzed by MEME (https://meme-suite.org/meme/tools/meme, accessed on 24 December 2021), and 10 conserved motifs were identified (Figure 2B). The details of these motifs are shown in Table S2. Among the 10 motifs, motifs 1, 2, and 6 conformed to the sequence characteristics of *C2H2-Zinc finger*. As a result, only 145 members had any of motifs 1, 2, and 6 left, although we identified 165 members based on the sequence alignment. In addition, motif 1 was distributed in nearly all of the *SbCsC2H2-ZFPs*, which implied that motif 1 could be a conserved and important motif among *C2H2-ZFPs* in *S. bicolor*. Motif 1 and motif 2 have the sequence “QALGGH”, the symbol of Q-type *C2H2-ZFPs*, which are specific to plants [38]. Motif 6 was mainly identified in clade A-I, A-V, C-II, and C-III, and motif 7 existed in clade C-II. Moreover, motif 9 and motif 10 were commonly identified in clade A-IV. In addition, there were the most motifs in clade C-I, including motifs 5, 1, 3, 2, 4, and 8, which implied that specific motifs might enable *SbC2H2-ZFPs*-specific functions. Overall, sequences with similar motif structures were clustered together, indicating the reliability of phylogenetic tree classification.

Furthermore, in order to understand the characteristics of *SbC2H2-ZFPs*, we analyzed their gene structures, including the number of introns and exons. In general, there were more exons than introns (Figure 2C). Among the 145 *SbC2H2-ZFPs*, a total of 79 members did not contain introns, accounting for 54.48%; a total of 38 members (26.21%) had one or two introns; a total of 28 members (19.31%) gained more than two introns. In all subclasses, there were members that contained introns and those that did not contain introns. Overall, the number of introns varied greatly in different *SbC2H2-ZFPs*, reflecting their diversity in structure and function. The details are listed in Table S1.

### 2.4. Chromosomal Distribution and Gene Duplication in SbC2H2-ZFP Genes

**Figure 3:**
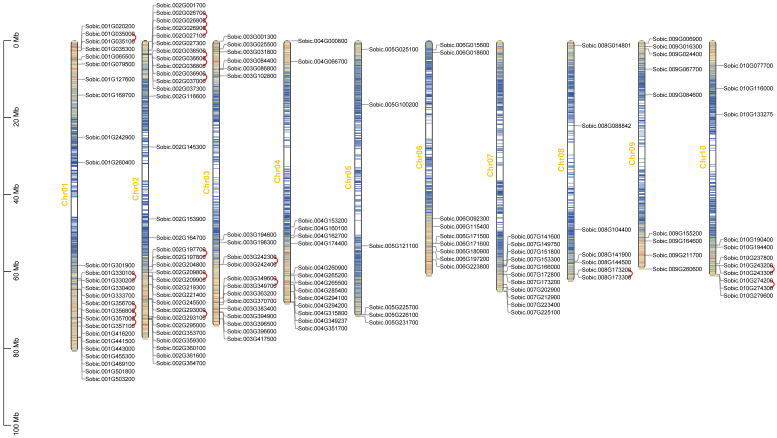
Chromosomal location of *SbC2H2-ZFP* genes in the *S. bicolor* genome. The length of chromosomes is measured in Mb. Genes of SbC2H2-ZFP are marked in black. Tandemly duplicated genes are represented with red wavy lines.

Chromosomal distribution of *SbC2H2-ZFP* genes was constructed according to *S. bicolor* genome information (Figure 3, Tables S1 and S3). There were *C2H2-ZFP* genes on each chromosome. Chr2 harbored the most *SbC2H2-ZFP* genes (32 genes, ~22.07%), followed by Chr1 (26, ~17.93%), while Chr5 contained the least (6 genes, ~4.14%). Chr3, Chr4, and Chr8 contained 19 (~13.10%), 15 (~10.34%), and 7 (~4.83%) *SbC2H2-ZFP* genes, respectively. Chr7 and Chr10 contained the same number of *SbC2H2-ZFP* genes (11 each, ~7.59%); furthermore, Chr6 and Chr9 each contained 9 (~6.21%) *SbC2H2-ZFP* genes. Moreover, if two genes were situated in the same chromosome within 100 kb of distance and separated by five or fewer genes, they would be regarded as tandemly duplicated genes [38]. As a result, we identified 19 tandem duplication events containing 33 *SbC2H2-ZFP* genes on Chr1, 2, 3, 8, and 10 (Figure 3). Interestingly, some SbC2H2-ZFPs participated in more than one tandem repeat event, such as Sobic.001G356800, Sobic.001G357000, Sobic.002G026800, Sobic.002G026900, and Sobic.002G036600. Moreover, all genes in tandem repeat events came from the same subfamily, suggesting the accuracy in the subfamily classification of the evolutionary tree (Figure 3, Table S3).

**Figure 4:**
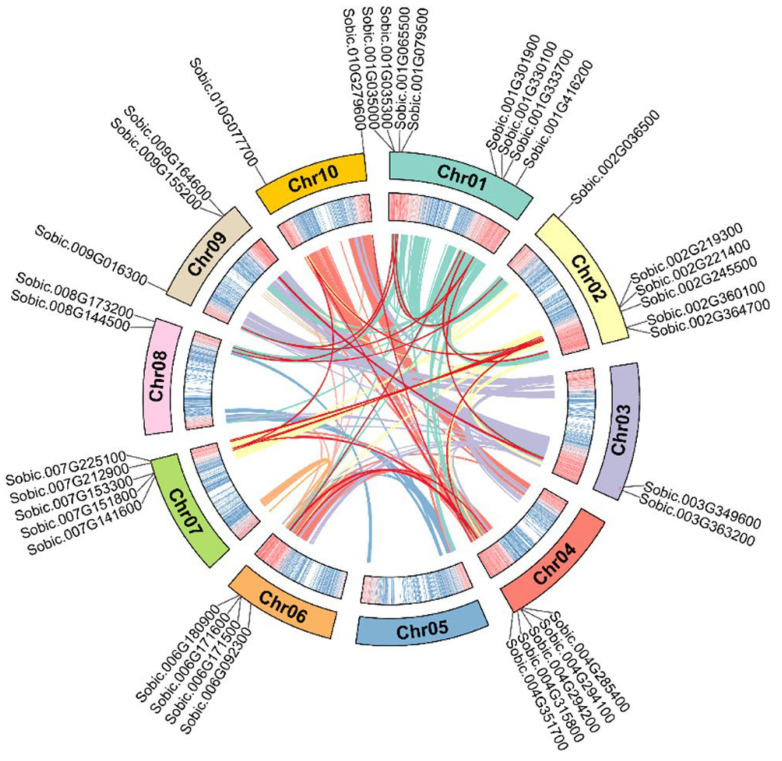
A schematic diagram of the synteny relationship of the *SbC2H2-ZFP* genes. Colors represent all collinear segments in the *S. bicolor* genome, and red lines represent duplicated *C2H2-ZFP* gene pairs. The outermost circle shows the chromosome number, and the second outer circle shows the density of each chromosome.

Apart from that, we found 24 segmental duplication events involving 34 *SbC2H2-ZFP* genes (Figure 4, Table S4). The *SbC2H2-ZFP* genes were distributed in 10 linkage groups (LGs). LG01 had the largest number of *SbC2H2-ZFP* genes (8, ~23.53%), followed by LG02 (6, ~17.65%), whereas LG05 did not contain any *SbC2H2-ZFP* genes. LG04, LG06, LG07, and LG09 contained 5 (~14.71%), 4 (~11.76%), 5 (~14.71%), and 3 (~8.82%) *SbC2H2-ZFP* genes, respectively. Moreover, LG03, LG08, and LG10 contained the least *SbC2H2-ZFP* genes (2 each, ~5.88%). In addition, out of all identified *SbC2H2-ZFP* genes, clade E had the most linked genes (25/34, ~73.53%).

### 2.5. Synteny Analysis of SbC2H2-ZFP Genes

**Figure 5:**
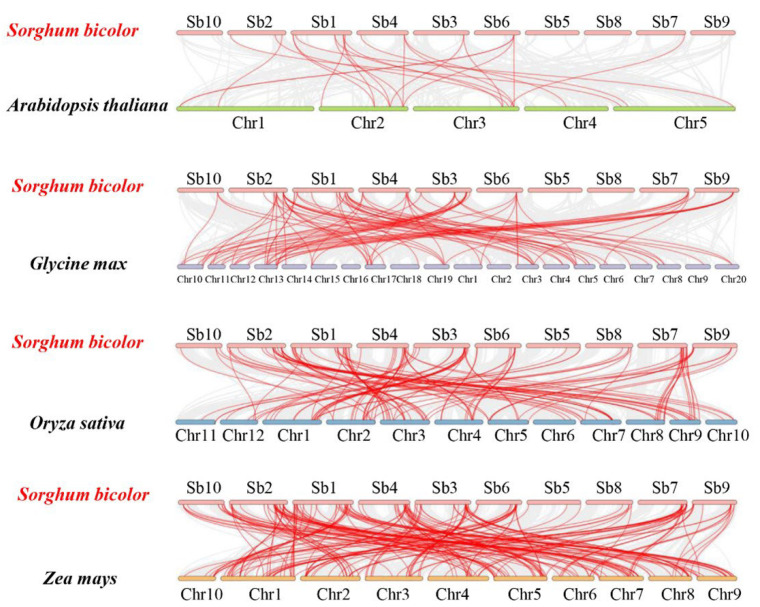
Synteny analyses of *C2H2-ZFP* genes between *S. bicolor* and four representative species. Gray lines represent the collinear regions within *S. bicolor* and other genomes, and red lines indicate the syntenic *SbC2H2-ZFP* gene pairs.

To further investigate evolution mechanisms of *SbC2H2-ZFP* genes, we analyzed syntenic relationships of *S. bicolor* with four representative species: two dicotyledon plant species (*A. thaliana* and *G. max*), and two monocotyledon plant species (*O. sativa* and *Z. mays*) (Figure 5, Table S5). A total of 110 *SbC2H2-ZFP* genes were syntenic with those in *A. thaliana* (14), followed by *G. max* (60), *O. sativa* (88), and *Z. mays* (148) (Table S5). The numbers of orthologous gene pairs between sorghum and the other four species (*A. thaliana*, *G. max*, *O. sativa*, and *Z. mays*) were 23, 85, 123, and 202, respectively.

Some *SbC2H2-ZFP* genes were associated with more than four syntenic gene pairs between *S. bicolor* and *Z. mays*, such as *Sobic.001G416200*, *Sobic.002G036500*, *Sobic.002G219300*, *Sobic.004G315800*, and *Sobic.007G151800*. This may indicate that before ancestors diverged, these orthologous gene pairs had already existed, suggesting that these genes are vital to the evolution of the C2H2-ZFP gene family. Apart from that, there were 55 gene pairs identified between *S. bicolor* and the other two monocotyledonous plants not existing between *S. bicolor* and two dicotyledonous plants, such as *Sobic.002G360100*, *Sobic.006G115400*, and *Sobic.001G503200*. As a result, these gene pairs may have formed after the divergence of monocots and dicots (Table S5).

### 2.6. Expression Patterns of SbC2H2-ZFPs in Several Tissues

**Figure 6:**
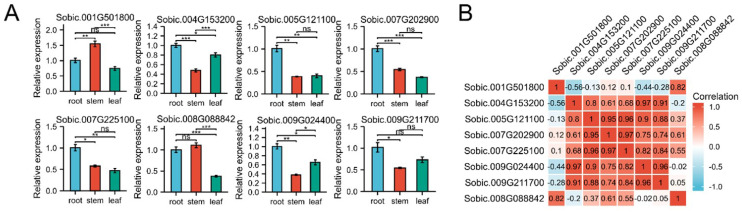
Tissue-specific expression of 8 *SbC2H2-ZFP* genes and the relevance of their expression. (**A**) Expression patterns of 8 *SbC2H2-ZFP* genes in root, stem, and leaf were analyzed by qRT-PCR. Error bars are obtained from three biological replicates, and standard error is selected as the value of the bar. Asterisks display significant expression differences of genes in different organs (* *p* < 0.05, ** *p* < 0.01, *** *p* < 0.001; one-way ANOVA). (**B**) Positive numbers: positive correlations; negative numbers: negative correlations.

To study the potential functions of *SbC2H2-ZFP* genes, we randomly selected one gene in each clade and analyzed their expression in three vegetative organs (roots, stems, leaves) by qRT-PCR (Figure 6A). Different *SbC2H2-ZFP* genes had different expression patterns in different organs, whereas almost all genes were expressed in all tissues. Several genes had the highest expression in the roots, such as *Sobic.004G153200*, *Sobic.005G121100*, *Sobic.007G202900*, *Sobic.007G225100*, and *Sobic.009G024400*. However, *Sobic.001G501800* and *Sobic.008G088842* had the highest expression in the stems. This indicated that the transcriptional abundance of different *SbC2H2-ZFP* genes varied in different organs, implying that SbC2H2-ZFPs play various roles in the growth and development of sorghum. Interestingly, the expression of some genes in the organs was quite correlated to that of others, implying that they might have synergism. For example, the expression of *Sobic.005G121100*, *Sobic.007G202900*, and *Sobic.007G225100* was significantly positively correlated in the roots, and they were all highly expressed. However, they were significantly negatively correlated with *Sobic.001G501800*.

### 2.7. Expression Patterns of SbC2H2-ZFPs in Response to Cold and Drought Stress

**Figure 7:**
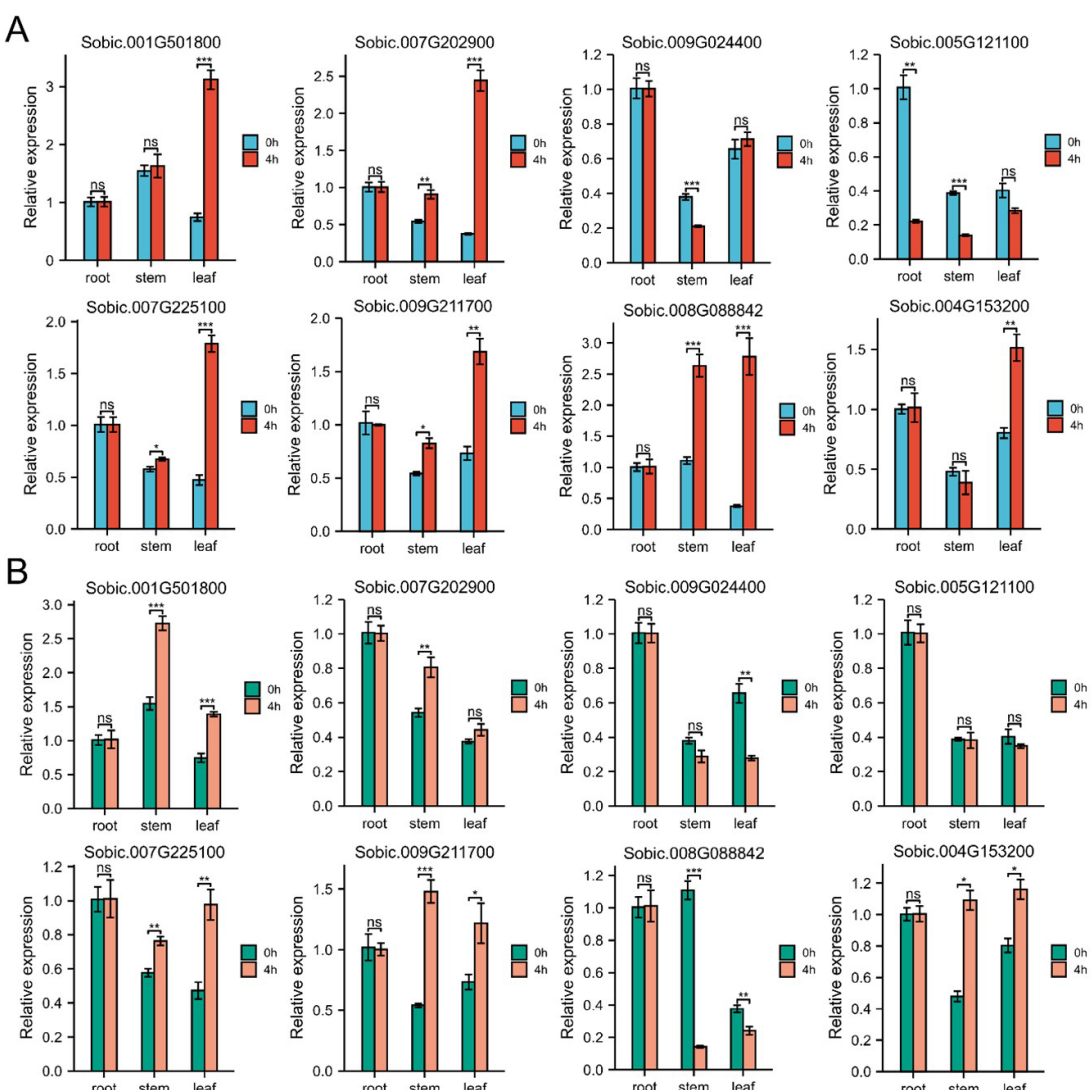
Expression of 8 *SbC2H2-ZFP* genes under cold (**A**) and PEG (**B**) stresses at the seedling stage. Error bars are obtained from three biological replicates, and standard error is selected as the value of the bar. Asterisks display significant expression differences of genes after treatment (* *p* < 0.05, ** *p* < 0.01, *** *p* < 0.001; *t*-test).

To analyze the potential roles of SbC2H2-ZFPs in sorghum responding to cold and drought stress, we performed qRT-PCR experiments under two abiotic stresses (Figure 7). As shown in Figure 7, some SbC2H2-ZFPs were significantly induced but others were extremely repressed. It was obvious that some SbC2H2-ZFPs showed various changes in different tissues and under different stresses. For example, under cold, the expression level of *Sobic.008G088842* increased in the stems and leaves, whereas it was down-regulated under drought stress. This indicated that *Sobic.008G088842* might be activated by cold but inhibited in drought. Expression of most genes in the roots was not affected by cold and drought stress, except *Sobic.005G121100*, which was significantly up-regulated under cold stress. Interestingly, a majority of genes was activated in the leaves under cold, such as *Sobic.001G501800*, *Sobic.007G202900*, *Sobic.007G225100*, *Sobic.009G211700*, *Sobic.008G088842*, and *Sobic.004G153200*, but more were up-regulated in the stems under drought.

## 3. Discussion

C2H2-type zinc finger proteins are one of the most abundant transcription factor families in higher plants. Previous reports indicate that they play an important role in cucumber, *Arabidopsis*, wheat, and tomato [39,40,41,42]. Therefore, many researchers have tried to perform genome-wide analysis on *C2H2-ZFPs* in various species, such as tomato, wheat, grape, and oyster mushroom [43,44,45,46], but little is known about sorghum C2H2-ZFP proteins. In this study, we carried out a genome-wide study of the *S. bicolor* C2H2-ZFP family and identified a total of 145 SbC2H2-ZFP members. Then we analyzed the evolutionary relationship of C2H2-ZFP between sorghum and *Arabidopsis* to infer the possible function of SbC2H2-ZFPs using a phylogenetic tree. What is more, motif composition, gene structure, chromosomal location, gene duplication events, and the expression of SbC2H2-ZFPs in different vegetative organs were analyzed, and their responses to cold and drought stress were investigated.

In this study, motifs 1, 2, and 6 were the characteristics of C2H2-zinc fingers. In addition to C2H2-type motifs, *SbC2H2-ZFPs* also contained many other motifs, suggesting that SbC2H2-ZFPs play an extensive role in higher plants. Motif 1 and Motif 2 had the sequence “QALGGH”, which is the symbol for the plant-specific Q-type C2H2-ZFPs [38]. Q-type C2H2-ZFPs have been reported to be involved in the growth, development, and organogenesis of a variety of plants, as well as in response to stresses and defense [47,48,49,50,51]. Motif 6 was mainly present in the clades A-I, A-V, C-II, and C-III, while motif 7 was present in clade C-II. Furthermore, motif 9 and motif 10 were common in the clade A-IV. Interestingly, most motifs were present in clade C-I, including motifs 5, 1, 3, 2, 4, and 8, implying that specific motifs may enable specific functions of SbC2H2-ZFPs.

Thirty-three *SbC2H2-ZFPs* (22.76%) were identified as tandem repeat genes, and 34 *SbC2H2-ZFPs* (23.45%) were identified as segmental repeat genes. Among them, several *SbC2H2-ZFPs* were involved in more than one tandem repeat event. These results suggested that gene duplication contributed to the expansion of a new gene family in the evolution of plant genome [52] and played a significant role in the evolution of the *SbC2H2-ZFP* genes.

The expression of a gene is often used to predict its function. Previous findings have shown that the expression of C2H2-ZFP genes was affected by tissue differences and various abiotic stresses [46,53]. Our results showed that among the selected SbC2H2-ZFPs, most genes were expressed in roots higher than those in leaves or stems, but almost all SbC2H2-ZFP members were expressed in roots, stems, and leaves. This indicated that the transcript abundances of different *SbC2H2-ZFP* genes were different in different organs, suggesting that SbC2H2-ZFP plays different roles in the growth and development of sorghum. It has been reported that plant growth and development are affected by the transcript abundance of *C2H2-ZFP* genes [26,54,55]. In addition, it was found that some SbC2H2-ZFPs showed different changes in different tissues or stresses by analyzing the expression of SbC2H2-ZFP under cold and drought stress. For example, under cold stress, the expression level of *Sobic.008G088842* was increased in stems and leaves, while it was down-regulated under drought stress. This indicated that the response of Sobic.008G088842 to different stresses might be worth further study. In addition, most genes were activated in leaves under cold stress, but more genes were up-regulated in stems under drought treatment. As a result, sorghum leaves may be suitable materials to study cold stress in the future, but stems might be better for drought stress research.

## 4. Materials and Methods

### 4.1. Identification of C2H2 ZFPs in Sorghum bicolor

The *S. bicolor* genome sequence was downloaded from the Phytozome v13 database (https://phytozome-next.jgi.doe.gov/info/Sbicolor_v3_1_1, accessed on 5 September 2021) [56]. The protein sequences of C2H2 ZFPs in Arabidopsis were downloaded from TAIR (https://www.arabidopsis.org/servlets/Search?search_action=sendToSequenceDLAll&type=gene&pageNum=0&size=4&query_id=41631445, accessed on 5 September 2021). All possible C2H2 ZFPs in *S. bicolor* were identified according to HMM profiles including PF00096, PF13894, PF13912, PF18414, PF16622, and PF18658 by using TBtools [57]. After this step, we obtained 184 *S. bicolor* C2H2-ZFPs. Then, all potential protein sequences were submitted to the *NCBI-BLAST* database (https://blast.ncbi.nlm.nih.gov/Blast.cgi?PROGRAM=blastp&PAGE_TYPE=BlastSearch&LINK_LOC=blasthome, accessed on 25 September 2021) and were validated through *UniprotKB/Swiss-Prot(swissprot)* and *MEME*. Eventually, 145 *S. bicolor* C2H2-ZFPs were reserved.

### 4.2. C2H2-ZFP Gene Structure and Conserved Motifs

To calculate molecular weights (Mws) and theoretical isoelectric points (pIs) of SbC2H2-ZFPs, all gene sequences were submitted to ExPASy (http://web.expasy.org/, accessed on 30 September 2021). Moreover, the gene structure of *SbC2H2-ZFPs* was obtained using the “Gene Location Visualize (Advanced)” function in TBtools. Apart from that, we used MEME 5.4.1 (https://meme-suite.org/meme/tools/meme, accessed on 24 December 2021) to analyze the motifs of SbC2H2-ZFP proteins. The parameters we used were as follows: motif sites distribution, zero or one occurrence per sequence; the maximum number of motifs, 10; minimum sites of each motif, 21; maximum sites of each motif, 30 [58].

### 4.3. Chromosomal Location and Gene Duplication of C2H2-ZFPs in S. bicolor

The chromosomal locations of *SbC2H2-ZFPs* were visualized by MCScanX and TBtools [57,59]. The default e-value cutoff of MCScanX is 1 × e^−10^. The origin of C2H2-ZFP members was analyzed by MCScanX with default parameters. Furthermore, if two genes were located in the same chromosome within 100 kb of distance, and separated by five or fewer genes, they would be identified as tandemly duplicated genes [38]. Apart from that, we studied *C2H2-ZFP* homology between *S. bicolor* and four other plants (*A. thaliana*, *O. sativa subsp. indica*, *Z. mays*, and *G. max*) by Dual Synteny Plotter [57]. The genome sequences of four species were downloaded from the Phytozome database (https://phytozome-next.jgi.doe.gov/, accessed on 30 December 2021) [60,61,62,63,64].

### 4.4. Phylogenetic Analysis of C2H2-ZFPs in S. bicolor

The full-length protein sequences of *S. bicolor* and *A. thaliana* C2H2-ZFPs were used for phylogenetic analysis. Multiple sequence alignments were performed with MUSCLE (https://www.ebi.ac.uk/Tools/msa/muscle/, accessed on 25 October 2021), and the resulting sequences were trimmed using trimAl. Then, a phylogenetic tree was inferred using IQ-TREE with a bootstrap value of 1000. Apart from that, the phylogenetic tree was visualized and annotated by iTOL (https://itol.embl.de/, accessed on 30 October 2021).

### 4.5. Plant Materials and Abiotic Stress in S. bicolor

*Sorghum bicolor* BTx623 was used in this study. *S. bicolor* was grown in uniformly mixed Pindstrup substrate (www.pindstrup.com, accessed on 30 October 2021) [65] in a light incubator with a 16 h/30 °C day and 8 h/25 °C night regime. The roots, stems, and leaves from five plants were collected, quickly placed in liquid nitrogen, and stored at −80 °C until further use. Apart from that, sorghum plants at 40 days were selected for drought by treating with 10% PEG6000 for 4 h and cold stress by being placed in 4 °C for 4 h. Each treatment had five replicates, and all samples collected were stored at −80 °C.

### 4.6. Total RNA Extraction and qRT-PCR Analysis

Total RNA of *S. bicolor* samples was extracted using a Plant RNA Kit (Omega Bio-tek Inc., Norcross, GA, USA) and reverse transcribed by a PerfectStart Uni RT&qPCR Kit (Transgen Biotech, Beijing, China). Specific primers were designed by Oligo 7.0 (Table S6). The qRT-PCR was conducted, and each selected gene was assayed at least three times. We used the EIF4α (eukaryotic initiation factor 4-α) gene as the control, the expression of which was stable in almost all growth stages and tissues [66]. The expression data were calculated according to the 2−(ΔΔCT) method and visualized using R 3.6.3 with the ggplot2 package (Version 3.3.5, Wickham, 2016, https://cran.r-project.org/web/packages/ggplot2/index.html, accessed on 5 September 2021).

## 5. Conclusions

We identified 145 C2H2-ZFP members that were randomly distributed on 10 chromosomes in *S. bicolor*. These members were divided into 11 clades based on the phylogenetic tree, and the genes in the same clade contained similar intron/exon and motif patterns. Furthermore, thirty-three tandem duplicated *SbC2H2-ZFPs* and 24 pairs of segmental duplicated genes were identified. Moreover, synteny analysis showed that sorghum had more collinear regions with monocotyledonous plants such as maize and rice than with dicotyledonous plants such as soybean and Arabidopsis. In addition, qRT-PCR analysis showed that several genes had the highest expression in the roots, such as *Sobic.004G153200* and *Sobic.005G121100*, while *Sobic.001G501800* and *Sobic.008G088842* had the highest expression in the stems. The experiment was also helpful for understanding the mechanisms of how C2H2-ZFPs regulated sorghum resistance to cold and drought stresses. For example, Sobic.008G088842 may play an important role in sorghum resistance to cold stress, while Sobic.004G153200 may improve drought tolerance. In conclusion, it provided important information for further study of the C2H2-ZFP family and a framework for stress-resistance research in sorghum.

## Data Availability

The *Sorghum bicolor* whole genome sequence information is from the Phytozome v13 database (https://phytozome-next.jgi.doe.gov/info/Sbicolor_v3_1_1, accessed on 5 September 2021). The *Sorghum bicolor* materials (DALISHI) used in this study were purchased from ChangJingZhongYe company (https://www.cmeii.com/, accessed on 20 September 2021). The datasets supporting the conclusions of this article are included in the article and its Supplementary Materials.

## Supplementary Materials

The following supporting information can be downloaded at: https://www.mdpi.com/article/10.3390/ijms23105571/s1.
